# Protective Antioxidant Effects of *Ganoderma lucidum* Against Prenatal Chlorpyrifos-Induced Developmental Nephrotoxicity in Rats

**DOI:** 10.3390/biomedicines14030658

**Published:** 2026-03-13

**Authors:** Şeyma Şimşirgil Kara, Dilek Sağır

**Affiliations:** Faculty of Health Sciences, Sinop University, Sinop 57000, Türkiye; ssimsirgil@sinop.edu.tr

**Keywords:** chlorpyrifos, *Ganoderma lucidum*, oxidative stress, antioxidant defense, apoptosis, developmental nephrotoxicity

## Abstract

**Background/Objectives:** Chlorpyrifos (CPF), a widely used organophosphate pesticide, has been associated with oxidative stress-mediated renal injury. Prenatal exposure may pose a risk for developmental nephrotoxicity; however, data regarding protective natural agents remain limited. This study evaluated the protective effects of *Ganoderma lucidum* (GNL) against CPF-induced renal alterations in rat offspring. **Methods:** Pregnant rats received CPF (5 mg/kg) and/or GNL (400 mg/kg) orally throughout gestation. On postnatal day 28, blood and kidney tissues from male offspring were collected for biochemical, ELISA, histopathological, immunohistochemical, and stereological analyses. **Results:** Prenatal CPF exposure significantly elevated serum urea and creatinine levels and induced oxidative stress, evidenced by increased malondialdehyde (MDA) and nitric oxide (NO) levels and decreased antioxidant enzyme activities (Superoxide dismutase (SOD), catalase (CAT), glutathione peroxidase (GPx), and reduced glutathione (GSH)) (all *p* < 0.05). Renal TNF-α and IL-6 levels were significantly increased, indicating inflammatory activation. Apoptotic signaling was enhanced, demonstrated by elevated cleaved caspase-3 levels and an altered Bax/Bcl-2 ratio. Tubular injury biomarkers, kidney injury molecule-1 (KIM-1) and neutrophil gelatinase-associated lipocalin (NGAL), were markedly increased. Histopathological findings revealed tubular degeneration, while stereological analysis confirmed significant increases in cortical and glomerular volumes. GNL co-treatment attenuated oxidative stress, suppressed inflammatory cytokines, reduced caspase-3 activation, lowered KIM-1 and NGAL levels, and preserved renal structure. **Conclusions:** Prenatal CPF exposure induces developmental nephrotoxicity through interconnected oxidative, inflammatory, and apoptotic mechanisms. *Ganoderma lucidum* mitigates these alterations by restoring antioxidant defense systems, modulating the Bax/Bcl-2 apoptotic balance, suppressing pro-inflammatory cytokine production, reducing tubular injury markers, and normalizing stereologically detected renal structural changes.

## 1. Introduction

Chlorpyrifos (CPF) (O,O-diethyl O-3,5,6-trichloro-2-pyridyl phosphorothioate) is a widely used organophosphate insecticide with well-documented toxicological effects. Following metabolic activation, CPF inhibits acetylcholinesterase (AChE), leading to excessive cholinergic stimulation and the generation of reactive oxygen species (ROS). The resulting oxidative stress contributes to cellular dysfunction and tissue injury. Increasing evidence indicates that CPF exposure induces neurotoxic, hepatotoxic, and nephrotoxic manifestations in both humans and experimental animal models [1,2,3].

Renal toxicity associated with CPF has been characterized by glomerular degeneration, tubular necrosis, and fibrotic alterations in experimental studies [4,5,6]. Elevated serum urea and creatinine levels further indicate impaired renal function. Oxidative stress is considered a central mechanism underlying CPF-induced renal injury, with increased malondialdehyde (MDA) and ROS levels accompanied by weakened endogenous antioxidant defenses [7,8]. Disruption of mitochondrial membrane potential and alterations in the Bax/Bcl-2 balance have been associated with apoptotic activation in renal tissue [9]. These findings highlight oxidative stress-mediated apoptosis as a key contributor to pesticide-related kidney injury.

The prenatal period represents a critical window of organogenesis during which nephrogenesis and glomerular maturation are highly susceptible to environmental insults [10,11]. Disruption of renal developmental programming during this stage may result in persistent structural and functional alterations extending into postnatal life [12]. Therefore, evaluating CPF-induced renal toxicity within a developmental framework is of particular toxicological relevance.

Beyond oxidative stress, accumulating evidence suggests that organophosphate-induced redox imbalance can amplify inflammatory signaling in renal tissue. Experimental studies have demonstrated that organophosphate pesticides such as methyl parathion induce renal inflammatory responses accompanied by increased TNF-α expression [13]. Organophosphorus compounds have also been described as modulators of inflammatory pathways, supporting their role in cytokine-mediated renal injury [1]. Similarly, pesticide-induced nephrotoxicity models, including fenamiphos exposure, have reported significant alterations in inflammatory markers together with oxidative and structural renal damage [14].

In parallel, tubular injury biomarkers such as kidney injury molecule-1 (KIM-1) and neutrophil gelatinase-associated lipocalin (NGAL) have emerged as sensitive indicators of early tubular epithelial damage. These markers increase in response to renal insult and correlate with histopathological and functional indices of tubular injury, often preceding changes in traditional parameters such as serum creatinine [15,16]. Accordingly, the integrated evaluation of oxidative stress, inflammatory mediators, apoptotic signaling, and tubular injury biomarkers may provide a more comprehensive understanding of pesticide-induced renal toxicity.

*Ganoderma lucidum* (GNL), commonly known as “Reishi,” has attracted considerable pharmacological interest due to its antioxidant, anti-inflammatory, and cytoprotective properties [17,18]. Its bioactive components, including triterpenoids and polysaccharides, have been shown to reduce lipid peroxidation, enhance antioxidant enzyme activities, and attenuate inflammatory and apoptotic responses in experimental kidney injury models [19,20]. These findings suggest that GNL may represent a promising natural candidate for mitigating pesticide-induced renal damage.

However, although CPF-induced renal toxicity has been investigated in adult exposure models, limited data are available regarding its developmental impact following prenatal exposure. Moreover, no study to date has comprehensively integrated biochemical, inflammatory, apoptotic, tubular injury, and stereological analyses to evaluate the protective role of *Ganoderma lucidum* in a prenatal CPF-induced nephrotoxicity model.

The present study was therefore designed to investigate oxidative stress, inflammatory activation, apoptotic signaling, tubular injury, and structural alterations in the kidneys of offspring following prenatal CPF exposure, and to evaluate the potential protective effects of *Ganoderma lucidum* against these toxic changes.

## 2. Materials and Methods

### 2.1. Animals and Experimental Design

For this study, 21 female and 21 male Wistar albino rats (210–250 g) were obtained from the Ondokuz Mayıs University Laboratory Animal Application and Research Center for mating purposes. For the purpose of mating, a total of four rats, two females and two males, were placed within the same cage. The presence of the vaginal plug was widely accepted as an indication of pregnancy, and the day of its observation was designated as the first day of pregnancy. The pregnant rats were maintained in standard, sterile plastic cages, with a humidity level of 55–60% and a room temperature of 19–22 °C. The animals were provided with ad libitum (unrestricted) access to feed and tap water.

The pregnant rats were randomly divided into five groups: Control (C), Chlorpyrifos (CPF), Chlorpyrifos + *Ganoderma lucidum* (CPF + GNL), *Ganoderma lucidum* (GNL), and Sham. Chlorpyrifos (Sigma Aldrich, Bayswater, Australia) was administered at a dose of 5 mg/kg [4], and *Ganoderma lucidum* extract (in the commercial form) was administered at a dose of 400 mg/kg [21] daily via oral gavage from the first day of pregnancy until birth. No procedure was performed on the rats in the control group, while those in the Sham group were given corn oil, the carrier substance in which the drugs were dissolved, using the same method.

The selected dose of chlorpyrifos (CPF; 5 mg/kg) was based on previous experimental studies demonstrating significant biochemical and histopathological alterations at subchronic exposure levels without overt systemic toxicity, including documented renal structural changes in rodent models [6]. This dose has been widely used to model low-to-moderate environmental exposure conditions in vivo. The *Ganoderma lucidum* (GNL) dose of 400 mg/kg was chosen according to prior preclinical studies reporting systemic antioxidant and protective effects at this pharmacologically active and well-tolerated dose in rodents. Administration of GNL extract at 400 mg/kg has been shown to mitigate oxidative stress and inflammatory responses without evidence of toxicity [22,23]. Although direct interspecies extrapolation is limited, the selected doses align with established experimental paradigms of toxic exposure and therapeutic intervention, thereby supporting the translational relevance of the present study.

*Ganoderma lucidum* extract was obtained from a commercial supplier in powdered form and used as provided by the manufacturer. The extract was prepared from the fruiting bodies of *Ganoderma lucidum* using a hot water/dual extraction method with an extract ratio of 15:1. According to the manufacturer’s specifications, the extract contained approximately 40% polysaccharides, 30% beta-glucans, 5% terpenes, and 1% ganoderic acids. The remaining fraction consisted of natural mushroom-derived components. The extract was stored under recommended conditions and freshly prepared in appropriate solvent prior to administration. The reported bioactive composition was based on the manufacturer’s certificate of analysis and was not independently re-quantified in the present study.

Subsequent to spontaneous normal delivery, two male offspring were randomly selected from each female rat that gave birth to newborn (NB) rats, ensuring systematic random sampling in stereological analyses. Eight NB rats were selected from each group, resulting in a total of 40 male NB rats included in the study. The rats were housed under the same environmental conditions until the 28th day after birth (postnatal week 4), at which point they were used for experimental procedures.

The study protocol was approved by Ondokuz Mayıs University Experimental Animal Ethics Committee (Ethics Code: E-68489742-604.02-2500219669). All experimental procedures were performed in accordance with the relevant guidelines and regulations. The present study was conducted in accordance with the ARRIVE guidelines.

### 2.2. Collection of Blood and Tissue Samples

All rats were anesthetized with ketamine hydrochloride (40–50 mg/kg; Alfazyne^®^, Egevet, Kemalpaşa, Türkiye) and then sacrificed by intracardiac perfusion at room temperature. For the purpose of biochemical analysis, blood samples were collected from the hearts of anesthetized rats during perfusion. The samples were then subjected to centrifugation at 2000 rpm for 15 min at 4 °C (Hettich, Tuttlingen, Germany). Following this, the serum was separated, and the samples were stored at −80 °C.

Following this, both kidneys were promptly extracted from each animal, their weight was measured, and the total kidney weights were documented. The values were calculated as relative kidney weight relative to body weight. Tissue samples reserved for histological examination were fixed in 4% neutral buffered formaldehyde. Tissues reserved for biochemical analysis were stored in a deep freezer at −80 °C until analysis.

### 2.3. Determination of Serum Urea and Creatinine Levels

Serum urea levels were measured using the Urea Test Kit (ab83362, Abcam, Cambridge, UK), which employs a colorimetric probe that forms a stable chromophore detectable at 570 nm as a result of the enzymatic conversion of urea to ammonia [24]. Serum creatinine levels were determined using the Creatinine Test Kit (ab65340, Abcam, Cambridge, UK), which is based on the Jaffé alkaline picrate reaction that produces a stable chromophore measurable at 570 nm [25]. All measurements were performed in accordance with the manufacturer’s protocol, and the results were expressed in mg/dL.

### 2.4. Preparation of Kidney Homogenates for Biochemical Assays

Following the sacrifice procedure, kidney tissues were rapidly removed from the rats and washed with ice-cold phosphate-buffered saline (PBS, 0.1 M, pH 7.4) to remove blood and debris. After weighing each tissue, it was homogenized in cold PBS at a 1:10 (*w*/*v*) ratio using a Teflon-glass homogenizer on ice. The resulting homogenates were centrifuged at 4 °C at 10,000 rpm for 15 min to separate the supernatant. Protein levels were measured for the standardization of biochemical analyses. The procedure applied was adapted from methods described in previous studies [26,27].

### 2.5. Determination of Oxidative Stress Markers in Kidney Tissue

The levels of lipid peroxidation (MDA) in kidney tissues were measured using the TBARS method described by Ohkawa et al. (1979) [27]. The levels of nitric oxide (NO) were determined using the Griess reaction, as described by Green et al. (1982) [28]. The results were expressed as µmol/mg protein [27,28].

### 2.6. Antioxidant Enzyme Activities in Kidney Tissues

The measurement of superoxide dismutase (SOD) activity was conducted in accordance with the methodology established by Sun et al. (1988) [29]. Catalase (CAT) activity was measured in accordance with the method developed by Aebi (1984) [26]. Glutathione peroxidase (GPx) activity was measured according to the protocol outlined by Paglia and Valentine (1967) [30]. The determination of reduced glutathione (GSH) levels was performed in accordance with the method described by Beutler et al. (1963) [31]. The results are expressed in terms of U/mg protein for enzyme activity and µmol/mg protein for GSH levels [26,29,30,31].

### 2.7. Measurement of Inflammatory Cytokines

To evaluate inflammatory signaling, renal tumor necrosis factor-α (TNF-α) and interleukin-6 (IL-6) levels were measured using rat-specific sandwich ELISA kits (BT-Laboratory, Shanghai, China; TNF-α: Cat. No. E0764Ra; IL-6: Cat. No. E0135Ra) according to the manufacturer’s instructions.

The standard curve detection ranges were 5–1000 ng/L for TNF-α and 0.1–40 ng/L for IL-6. The reported assay sensitivities were 2.51 ng/L for TNF-α and 0.052 ng/L for IL-6. According to the manufacturer’s validation data, intra-assay coefficients of variation ranged between 4.6–5.7% for TNF-α and 4.5–6.5% for IL-6, while inter-assay coefficients of variation were <10% for both kits.

Optical density was measured at 450 nm using a microplate reader. All samples were assayed within the linear dynamic range of the respective standard curves. Following appropriate unit conversion, cytokine concentrations were normalized to tissue weight and expressed as pg/g tissue. All samples were analyzed in duplicate.

### 2.8. Determination of Apoptotic Markers

Apoptotic pathway activation was assessed by measuring renal protein levels of cleaved (active) caspase-3 using a rat-specific ELISA kit (BT-Laboratory, Shanghai, China, Cat. No. E1648Ra), according to the manufacturer’s instructions. Cleaved caspase-3 was quantified to specifically reflect execution-phase apoptosis rather than total caspase-3 expression.

The detection range of the assay was 0.05–10 ng/mL, with a reported sensitivity of 0.02 ng/mL. The intra-assay coefficient of variation ranged between 4.1–6.6%, and the inter-assay coefficient of variation was <10%, indicating acceptable analytical precision.

Optical density was measured at 450 nm. All samples were measured within the linear range of the standard curve and normalized to tissue weight. Results were expressed as ng/g tissue. Measurements were performed in duplicate.

### 2.9. Assessment of Renal Injury Biomarkers (KIM-1 and NGAL)

Renal KIM-1 and NGAL levels were measured using rat-specific sandwich ELISA kits (BT-Laboratory, Cat. Nos. E0549Ra and E0762Ra) according to the manufacturer’s protocols. These biomarkers were selected as sensitive indicators of tubular injury and early renal damage.

The detection ranges were 0.05–10 ng/mL for KIM-1 and 0.3–90 ng/mL for NGAL. The reported sensitivities were 0.01 ng/mL for KIM-1 and 0.15 ng/mL for NGAL. Intra-assay coefficients of variation ranged between 3.3–4.1% for KIM-1 and 3.4–7.9% for NGAL, with inter-assay coefficients of variation <10% for both assays.

Optical density was read at 450 nm using a microplate reader. All samples were analyzed within the linear dynamic range of the standard curves. Concentrations were calculated from the standard curves and normalized to total protein content determined by the Bradford method. Results were expressed as pg/mg protein. All measurements were performed in duplicate.

### 2.10. Histological Tissue Procedures and Analyses

After undergoing standard tissue processing procedures, fixed kidney tissues were embedded in paraffin blocks. Sections 5 µm thick were taken from the blocks using a rotary microtome. The kidney sections were stained with H&E for general morphological evaluation. The classic Masson’s trichrome protocol was performed to demonstrate collagen and the fibrous matrix. PAS staining was performed to demonstrate the basement membrane. The sections were examined and recorded using a digital video analysis system (Leica (Wetzlar, Germany) DFC450C and DM2500 with Leica Application Suite Version 4).

#### Histological Damage Scoring

Histopathological changes in kidney tissues were evaluated using Hematoxylin-Eosin (H&E)-stained sections. Three paraffin sections, spaced at least 100 µm apart and not adjacent to each other, were selected from each animal and examined under a light microscope. Five randomly selected microscopic fields were evaluated at 400× magnification in each section; a total of 15 fields per animal were analyzed. Tubular degeneration, epithelial shedding, interstitial edema, and inflammatory cell infiltration were evaluated using a semi-quantitative scoring system graded from 0 to 3: 0 = normal histological structure or no visible lesion; 1 = mild tubular dilatation, cellular swelling, or limited inflammation; 2 = moderate tubular necrosis, epithelial cell loss, and moderate inflammation; and 3 = widespread tubular necrosis, marked epithelial disruption, and intense inflammatory infiltration. The mean scores obtained for each animal were calculated to determine group averages and were then subjected to statistical analysis.

This method was applied with minor modifications based on previously defined semi-quantitative histopathological scoring systems [32,33].

### 2.11. Immunohistochemical Analysis

The Bax and B-cell lymphoma 2 (Bcl-2) immunohistochemical staining in kidney tissues was performed according to the previously described procedure [34], with minor modifications. Briefly, 5-µm-thick paraffin-embedded sections were deparaffinized and rehydrated, followed by heat-induced antigen retrieval in citrate buffer (10 mM, pH 6.0) at 95–98 °C for 15 min. Endogenous peroxidase activity was blocked with 3% hydrogen peroxide. Sections were incubated overnight at 4 °C with primary antibodies against Bax (SC-7480, Santa Cruz, CA, USA) and Bcl-2 (Santa Cruz, CA, USA) at a dilution of 1:100. After washing, slides were treated with a biotinylated secondary antibody and streptavidin–peroxidase complex. Immunoreactivity was visualized using 3,3′-diaminobenzidine (DAB), and all tissues were counterstained with Mayer’s hematoxylin. Negative control sections were processed by omitting the primary antibody. The preparations were examined and photographed under a light microscope (Leica, DFC 450C & DM 2500, Leica Application Suite Version 4).

In each section, the presence of immunohistochemical staining was examined in a minimum of five randomly selected areas at 400× magnification. The evaluation was conducted in a blind manner, with the researchers unaware of the group information. For each animal, a minimum of three non-adjacent sections (with a minimum interval of 100 µm) were utilized. In this study, a total of 21 glomeruli were evaluated for each animal, with seven glomeruli selected at random for analysis in each section.

Although CPF-induced renal injury is frequently associated with tubular alterations, glomerular apoptosis plays a critical role in nephron integrity and long-term renal function. Since our stereological analyses primarily focused on glomerular structural parameters, Bax and Bcl-2 expression was evaluated specifically within glomerular regions to ensure methodological consistency between structural and apoptotic assessments.

The assessment of immunoreactivity was conducted on the basis of prevalence and severity. The prevalence was scored as 0.1 for <25%, 0.4 for 26–50%, 0.6 for 51–75%, and 0.9 for 76–100%. The intensity of the pain was determined on a scale of 0 (none), 0.5 (very low), 1 (low), 2 (moderate), and 3 (severe). The histoscore was determined by employing the prevalence × intensity formula. Finally, immunoreactivity values for Bax and Bcl-2 were determined separately, and the Bax/Bcl-2 ratio was calculated on a glomerular basis [34].

### 2.12. Stereological Analysis

The estimation of cortex, medulla, and glomerulus volume ratios was performed using the Cavalieri principle’s point counting method [35]. Sections measuring 5 microns in thickness were obtained at 1/15 intervals from paraffin blocks in accordance with systematic random sampling protocols, yielding approximately 13–17 sections per kidney. The sections were stained with hematoxylin and eosin. To analyze the images, a digital video analysis system (Leica, DFC 450C & DM 2500, Leica Application Suite Version 4) was employed. Images were acquired at 5× magnification for cortex and medulla analysis and at 40× magnification for glomerulus analysis. To this end, two distinct point counting grids were meticulously prepared. The grids were randomly placed on the computer screen, and the points corresponding to the relevant structures in each section were counted. Sparse points were used to estimate the cortex and medulla volumes, while dense points were used to estimate the glomerulus volume.

The volume of each structure was calculated using the following formula:

V = t × (a/p) × P

V: Volume, t: Cross-section thickness, a/p: Area per point, P: Number of points corresponding to the relevant structure.

The total volume was found by adding the volumes obtained from all cross-sections:

Total volume = V1 + V2 + … + Vn

The stereological sampling parameters, including the number of analyzed sections per animal, total counted points per structure, and coefficient of error (CE) values, are summarized in Table 1.

### 2.13. Statistical Analysis

All statistical analyses were carried out using SPSS software (version 21.0, IBM Corp., Armonk, NY, USA). The distribution of the data was examined with the Shapiro–Wilk test to determine whether the variables met the assumptions of normality. For datasets showing a normal distribution, comparisons among groups were performed using one-way analysis of variance (ANOVA) followed by Bonferroni’s post hoc test. When the data did not satisfy the normality criterion, the Kruskal–Wallis test was applied, and pairwise comparisons were made using the Mann–Whitney U test with Bonferroni adjustment. A *p*-value less than 0.05 was considered to indicate statistical significance.

## 3. Results

### 3.1. Kidney and Relative Kidney Weights

When kidney weights and relative kidney weights were compared between groups, the relative kidney weight (%) was found to be 1.05 ± 0.05 in the control group, 1.67 ± 0.09 in the CPF-treated group, 1.27 ± 0.05 in the CPF + GNL group, 1.02 ± 0.06 in the GNL group, and 1.03 ± 0.07 in the SHAM group.

**Figure 1:**
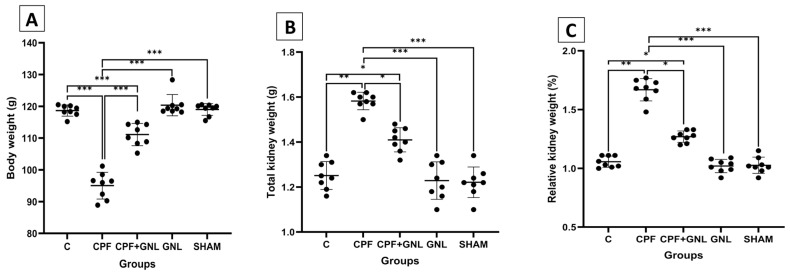
Body and kidney weight parameters in experimental groups. (**A**) Body weight, (**B**) total kidney weight, and (**C**) relative kidney weight (%) in Control (C), CPF, CPF + GNL, GNL, and Sham groups. Data are presented as mean ± SD with individual data points (*n* = 8 per group). Statistical differences between groups are indicated by brackets (* *p* < 0.05, ** *p* < 0.01, *** *p* < 0.001).

A statistically significant increase in relative kidney weight was observed in the CPF group compared to the control group (*p* < 0.01). In the CPF + GNL group, relative kidney weight was significantly decreased compared to the CPF group (*p* < 0.05), indicating a protective effect of GNL treatment. In addition, relative kidney weight in the CPF group was significantly higher than in the GNL and SHAM groups (*p* < 0.001). No significant differences were observed between the GNL or SHAM groups and the control group (*p* > 0.05) (Figure 1).

### 3.2. Biochemical Analysis Results

#### 3.2.1. Renal Function Parameters (Serum Urea and Creatinine Levels)

**Figure 2:**
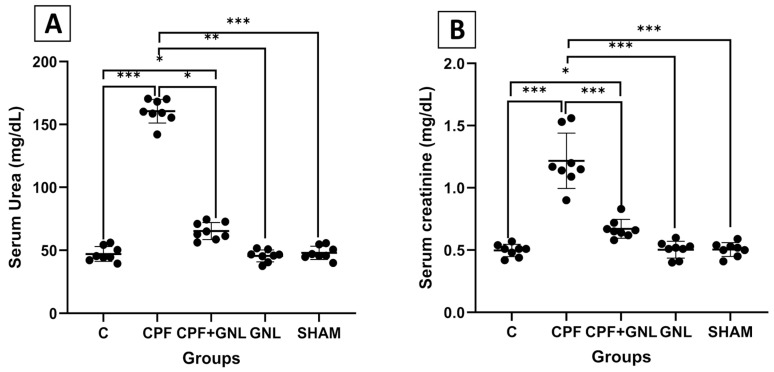
Renal function parameters in experimental groups. (**A**) Serum urea and (**B**) serum creatinine levels in Control (C), CPF, CPF + GNL, GNL, and Sham groups. Data are presented as mean ± SD with individual data points (*n* = 8 per group). Statistical differences between groups are indicated by brackets (* *p* < 0.05, ** *p* < 0.01, *** *p* < 0.001).

Serum urea and creatinine levels showed changes consistent with those observed in relative kidney weight (Figure 2). Both parameters were significantly increased in the CPF group compared with the control group (*p* < 0.001). Co-administration of GNL with CPF significantly reduced serum urea (*p* < 0.05) and creatinine (*p* < 0.001) levels compared with the CPF group. In addition, serum urea levels in the CPF + GNL group remained higher than those of the control group (*p* < 0.01). No significant differences were observed between the GNL or SHAM groups and the control group (*p* > 0.05).

#### 3.2.2. Oxidative Stress and the Antioxidant Defense System Markers

**Figure 3:**
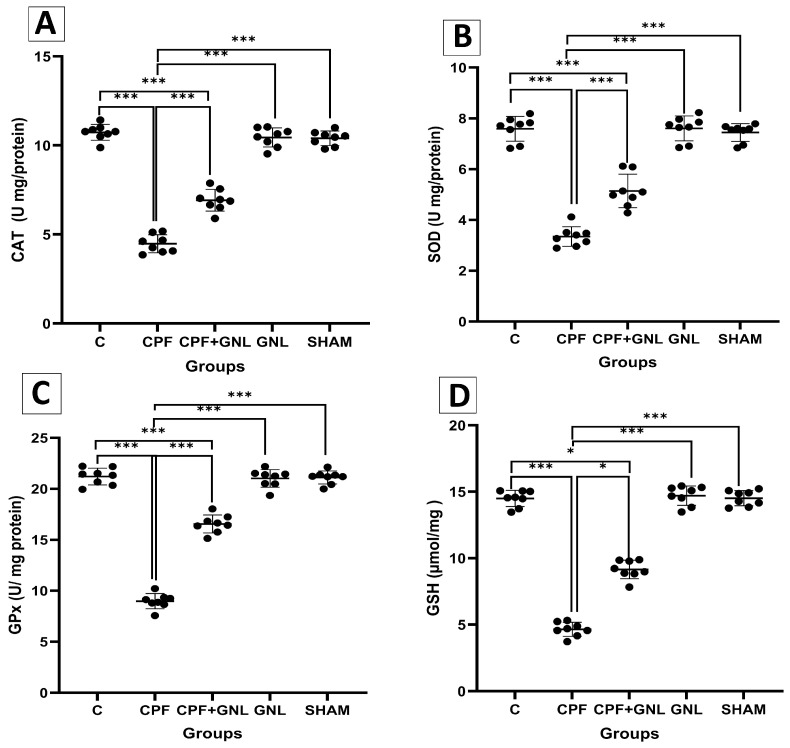
Serum antioxidant parameter levels by group: (**A**) CAT, (**B**) SOD, (**C**) GPx, (**D**) GSH, and levels in Control (C), CPF, CPF + GNL, GNL, and Sham groups. Data are presented as mean ± SD with individual data points (*n* = 8 per group). Statistical differences between groups are indicated by brackets (* *p* < 0.05, *** *p* < 0.001).

**Figure 4:**
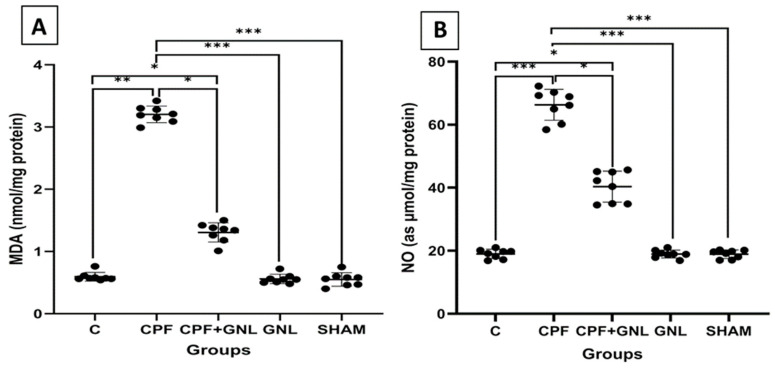
Oxidative stress markers in kidney tissue of experimental groups. (**A**) Malondialdehyde (MDA) and (**B**) nitric oxide (NO) levels in Control (C), CPF, CPF + GNL, GNL, and Sham groups. Data are presented as mean ± SD with individual data points (*n* = 8 per group). Statistical differences between groups are indicated by brackets (* *p* < 0.05, ** *p* < 0.01, *** *p* < 0.001).

Biochemical analyses demonstrated a marked disturbance in oxidative balance following CPF exposure (Figure 3 and Figure 4). MDA levels were significantly increased in the CPF group compared with the control group (*p* < 0.01). In the CPF + GNL group, MDA levels were significantly decreased compared with the CPF group (*p* < 0.05). In addition, MDA levels in the CPF group were significantly higher than those in the GNL and SHAM groups (*p* < 0.001).

Similarly, NO levels were significantly elevated in the CPF group compared with the control group (*p* < 0.001). Co-administration of GNL significantly reduced NO levels compared with the CPF group (*p* < 0.05), whereas NO levels in the CPF group remained significantly higher than those in the GNL and SHAM groups (*p* < 0.001).

Regarding antioxidant parameters, CAT, SOD, and GPx levels were significantly lower in the CPF group compared with the control group (*p* < 0.001). Co-treatment with GNL significantly increased CAT, SOD, and GPx levels compared with the CPF group (*p* < 0.001). GSH levels were also significantly decreased in the CPF group compared with the control group (*p* < 0.001), whereas GSH levels in the CPF + GNL group were significantly higher than those in the CPF group (*p* < 0.05). No significant differences were observed between the GNL or SHAM groups and the control group (*p* > 0.05).

#### 3.2.3. Renal Inflammatory Cytokine Profile

**Figure 5:**
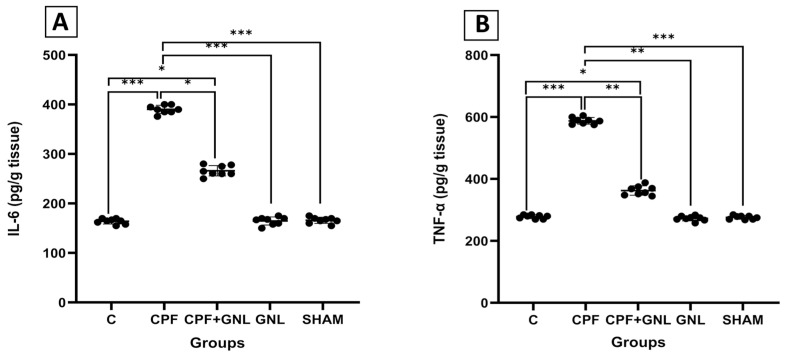
Inflammatory cytokine levels in kidney tissue of experimental groups. (**A**) IL-6 and (**B**) TNF-α levels in Control (C), CPF, CPF + GNL, GNL, and Sham groups. Data are presented as mean ± SD with individual data points (*n* = 8 per group). Statistical differences between groups are indicated by brackets (* *p* < 0.05, ** *p* < 0.01, *** *p* < 0.001).

Assessment of renal cytokine levels revealed a pronounced pro-inflammatory shift in the CPF group (Figure 5). IL-6 and TNF-α concentrations were significantly higher in the CPF group compared with the control group (*p* < 0.001). In the CPF + GNL group, cytokine levels were significantly reduced compared with the CPF group (IL-6: *p* < 0.05; TNF-α: *p* < 0.01), indicating attenuation of CPF-induced inflammatory signaling.

Furthermore, IL-6 and TNF-α levels in the CPF group were significantly higher than those observed in the GNL and SHAM groups (*p* < 0.001). In contrast, no significant differences were detected between the GNL or SHAM groups and the control group (*p* > 0.05), indicating that GNL alone did not induce inflammatory alterations (Figure 5).

#### 3.2.4. Cleaved Caspase-3 Protein Levels

**Figure 6:**
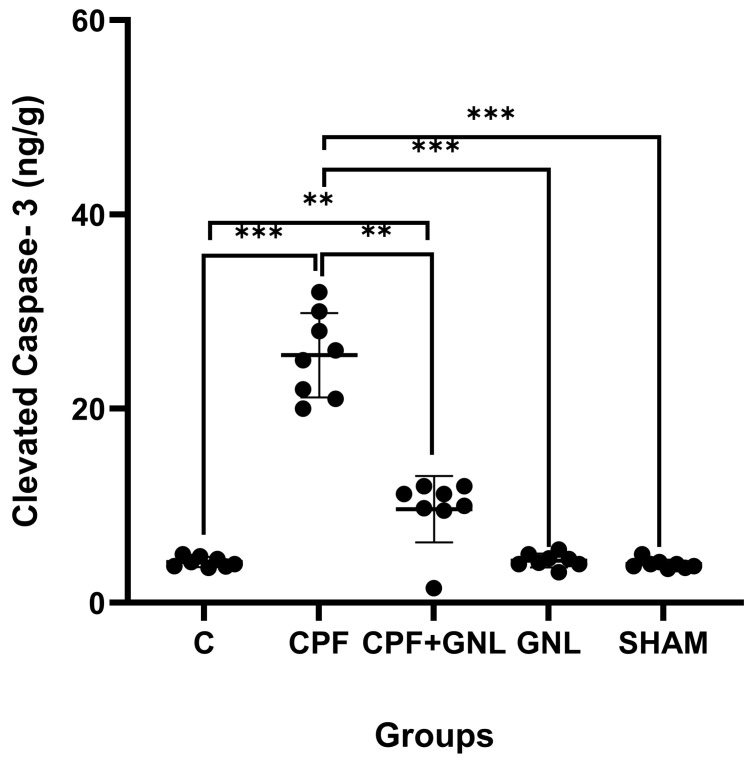
Apoptotic marker levels in kidney tissue of experimental groups. Cleaved caspase-3 levels in Control (C), CPF, CPF + GNL, GNL, and Sham groups. Data are presented as mean ± SD with individual data points (*n* = 8 per group). Statistical differences between groups are indicated by brackets (** *p* < 0.01, *** *p* < 0.001).

Prenatal CPF exposure markedly activated apoptotic signaling in renal tissue (Figure 6). Cleaved caspase-3 levels were significantly higher in the CPF group compared with the control group (*p* < 0.001). In the CPF + GNL group, cleaved caspase-3 levels were significantly reduced compared with the CPF group (*p* < 0.01), indicating attenuation of CPF-induced apoptotic activation. However, cleaved caspase-3 levels in the CPF + GNL group remained significantly higher than those of the control group (*p* < 0.01).

Furthermore, cleaved caspase-3 levels in the CPF group were significantly higher than those observed in the GNL and SHAM groups (*p* < 0.001). In contrast, no significant differences were detected between the GNL or SHAM groups and the control group (*p* > 0.05), indicating that GNL alone did not induce apoptotic activation in renal tissue (Figure 6).

#### 3.2.5. Expression of Tubular Injury Biomarkers (KIM-1 and NGAL)

Prenatal CPF exposure resulted in a marked elevation in renal KIM-1 levels compared to the control group (*p* < 0.05). KIM-1 concentrations in the CPF group were approximately five-fold higher than controls, indicating significant tubular epithelial injury at postnatal day 28. Similarly, renal NGAL levels were significantly increased in the CPF group relative to controls (*p* < 0.05), further confirming sustained tubular damage.

**Figure 7:**
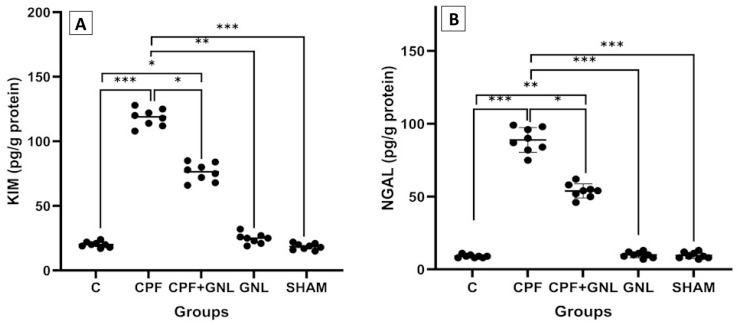
Renal injury biomarkers in kidney tissue of experimental groups. (**A**) KIM-1 and (**B**) NGAL levels in Control (C), CPF, CPF + GNL, GNL, and Sham groups. Data are presented as mean ± SD with individual data points (*n* = 8 per group). Statistical differences between groups are indicated by brackets (* *p* < 0.05, ** *p* < 0.01, *** *p* < 0.001).

Co-administration of GNL significantly reduced both KIM-1 and NGAL levels compared to the CPF group (*p* < 0.05). Although biomarker levels in the CPF + GNL group remained higher than controls, the reduction was statistically significant, suggesting partial attenuation of CPF-induced renal injury. Importantly, the GNL-alone and sham groups exhibited KIM-1 and NGAL levels comparable to the control group, indicating that GNL administration alone did not induce renal toxicity (Figure 7).

### 3.3. Histopathological Results

#### 3.3.1. General Histological Findings and Damage Scoring (H&E)

**Figure 8:**
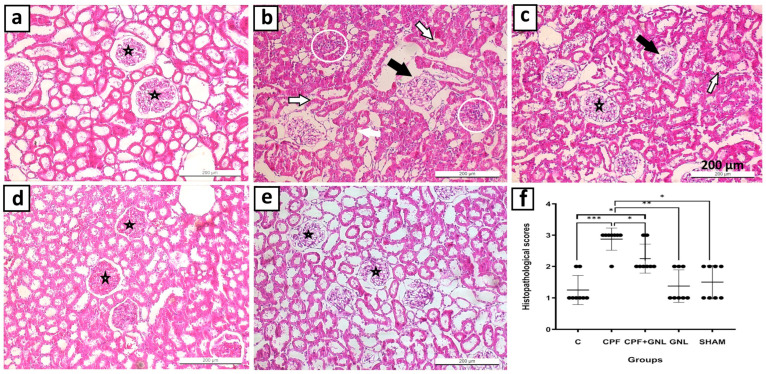
Representative hematoxylin–eosin (H&E) stained kidney sections from experimental groups: (**a**) control, (**b**) CPF, (**c**) CPF + GNL, (**d**) GNL, and (**e**) sham. Star: normal glomerulus; black arrow: degenerated glomerulus; white circle: inflammatory cell infiltration; white arrow: degenerated tubules. Scale bar = 200 µm; magnification ×200. (**f**) Histopathological injury scores in control (C), CPF, CPF + GNL, GNL, and sham groups. Data are presented as mean ± SD (*n* = 8). Statistical differences between groups are indicated by brackets (* *p* < 0.05, ** *p* < 0.01, *** *p* < 0.001).

Histological examination of kidney sections stained with hematoxylin-eosin (H&E) revealed normal renal structure in the cortical and medullary regions in the control and SHAM groups. The glomeruli had regular contours, the Bowman’s space was of normal width, and the tubular epithelial cells had a regular morphology. In the CPF-treated group, however, significant structural changes were detected. Glomerular shrinkage, expansion of the Bowman’s space, degeneration of tubular epithelial cells, vacuolization, lumen narrowing, and epithelial shedding were observed. Additionally, cellular infiltration and vascular congestion in the interstitial area were noted. In the group treated with GNL, renal structure was largely preserved, with regular glomerular morphology and minimal degeneration of tubular cells. In the CPF + GNL group, structural integrity was significantly preserved, with limited inflammation and epithelial damage (Figure 8a–e).

According to the histopathological injury scoring evaluation, renal damage scores were significantly higher in the CPF group compared with the control group (*p* < 0.001). In contrast, histopathological scores were significantly lower in the CPF + GNL and GNL groups compared with the CPF group (*p* < 0.05). Furthermore, no significant differences were observed between the control, GNL, and SHAM groups (*p* > 0.05) (Figure 8f).

#### 3.3.2. Masson Trichrome and PAS Staining Results

**Figure 9:**
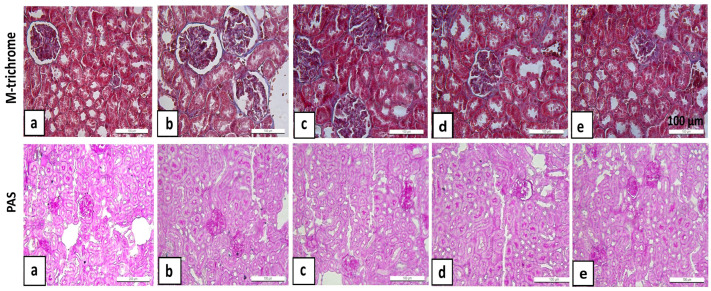
(**Upper panel**): Histological images of kidney tissue from all groups stained with Masson’s Trichrome stain. (**Lower panel**): Histological images of kidney tissue from the groups stained with PAS. (**a**) Control, (**b**) CPF, (**c**) CPF + GNL, (**d**) GNL, and (**e**) Sham groups. ×200.

In examinations performed with Masson Trichrome stain, normal collagen distribution was observed in kidney tissue in the control and sham groups. No significant areas of fibrosis were detected in the glomerular or tubular basement membranes. In the CPF-treated group, increased collagen accumulation in the interstitial areas and intensified fibrotic areas around the glomeruli and along the basement membrane were determined. In the GNL-treated group, collagen accumulation was minimal, and tissue organization was largely preserved. In the CPF + GNL group, fibrotic areas exhibited a significant reduction, and collagen distribution manifested characteristics analogous to those observed in the control group (Figure 9).

In PAS staining evaluations, it was determined that the glomerular basement membranes in the control and sham groups were smooth and thin in structure, and no thickening was observed in the tubular membranes. In the CPF group, thickening of the basement membranes, accumulation of PAS-positive material, and areas of intense staining around the glomeruli were observed. In the group receiving GNL, the structure of the basement membrane was largely preserved; in the CPF + GNL group, PAS staining was significantly reduced and membrane thickening was minimal (Figure 9).

#### 3.3.3. Immunohistochemical Analysis (Bax, Bcl-2, and Bax/Bcl-2 Ratio)

**Figure 10:**
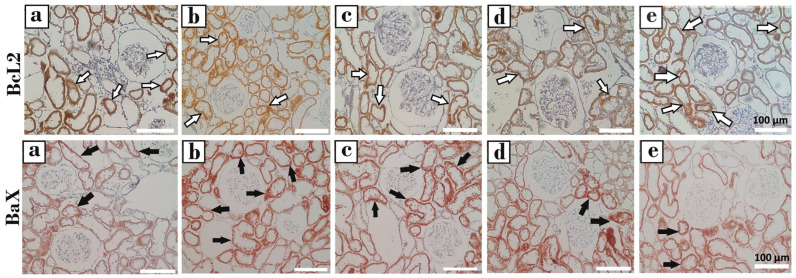
Images of kidney tissue from the Groups obtained by Bcl-2 immunohistochemical staining in the (**upper panel**). Images of kidney tissue from the groups obtained by Bax immunohistochemical staining in the (**lower panel**). White arrows: Bcl-2-positive cells; black arrows: Bax-positive cells. (**a**) Control, (**b**) CPF, (**c**) CPF + GNL, (**d**) GNL, and (**e**) Sham groups. DAB chromogen, counterstained with hematoxylin; ×20.

**Figure 11:**
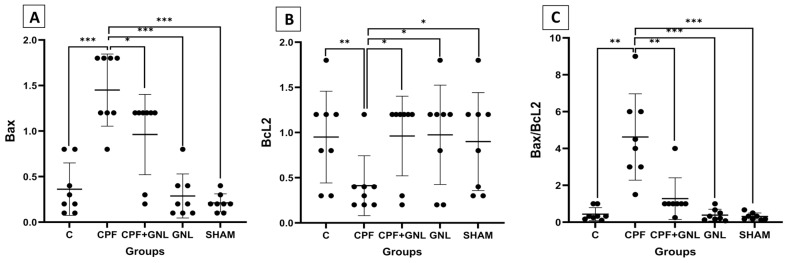
Apoptosis-related protein levels in kidney tissue of experimental groups. (**A**) Bax, (**B**) Bcl-2, and (**C**) Bax/Bcl-2 ratio in Control (C), CPF, CPF + GNL, GNL, and Sham groups. Data are presented as mean ± SD with individual data points (*n* = 8 per group). Statistical differences between groups are indicated by brackets (* *p* < 0.05, ** *p* < 0.01, *** *p* < 0.001).

Representative immunohistochemical staining images of Bax and Bcl-2 in kidney tissue are presented in Figure 10, while the quantitative immunoreactivity scores are shown in Figure 11. Bax immunoreactivity was significantly increased in the CPF group compared with the control group (*p* < 0.001). In contrast, Bax expression was significantly reduced in the CPF + GNL and SHAM groups compared with the CPF group (*p* < 0.001).

Conversely, Bcl-2 immunoreactivity was significantly decreased in the CPF group compared with the control group (*p* < 0.01). Treatment with GNL significantly increased Bcl-2 expression in the CPF + GNL, GNL, and SHAM groups compared with the CPF group (*p* < 0.05).

Accordingly, the Bax/Bcl-2 ratio was significantly higher in the CPF group compared with the control group (*p* < 0.01). In the CPF + GNL group, the Bax/Bcl-2 ratio was significantly reduced compared with the CPF group (*p* < 0.01). Furthermore, the Bax/Bcl-2 ratio in the CPF group remained significantly higher than those observed in the GNL and SHAM groups (*p* < 0.001).

### 3.4. Stereological Results

A comprehensive stereological analysis was performed on 13–17 systematically random sections from each animal to obtain mean values for cortex, medulla, and glomerular volumes.

Cortex volume was significantly increased in the CPF group compared with the control group (*p* < 0.01). In contrast, cortex volume was significantly reduced in the CPF + GNL group compared with the CPF group (*p* < 0.05). Moreover, cortex volume in the CPF group was significantly higher than those observed in the GNL and SHAM groups (*p* < 0.01).

No statistically significant differences in medulla volume were detected among the experimental groups (*p* > 0.05).

**Figure 12:**
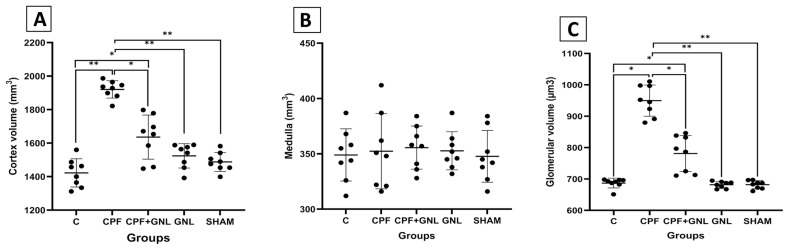
Stereological measurements of renal structural parameters in experimental groups. (**A**) Cortex volume, (**B**) medulla volume, and (**C**) glomerular volume in Control (C), CPF, CPF + GNL, GNL, and Sham groups. Data are presented as mean ± SD with individual data points (*n* = 8 per group). Statistical differences between groups are indicated by brackets (* *p* < 0.05, ** *p* < 0.01).

Similarly, glomerular volume was significantly increased in the CPF group compared with the control group (*p* < 0.05). In the CPF + GNL group, glomerular volume was significantly reduced compared with the CPF group (*p* < 0.05). Furthermore, glomerular volume in the CPF group remained significantly higher than those in the GNL and SHAM groups (*p* < 0.01) (Figure 12).

## 4. Discussion

Chlorpyrifos (CPF), a widely used organophosphate pesticide, has attracted increasing attention due to its developmental toxicity. Prenatal exposure to CPF has been reported not only to induce neurodevelopmental impairments but also to compromise the structural and functional integrity of vital peripheral organs, including the kidney [36]. Experimental evidence indicates that CPF-induced oxidative stress may result in long-lasting renal damage, characterized by enhanced lipid peroxidation and suppression of antioxidant defense mechanisms [37]. Despite these findings, studies specifically addressing the effects of prenatal CPF exposure on kidney development remain limited [36], highlighting a critical gap in the literature.

To address this deficiency, the present study comprehensively evaluated biochemical, histological, immunohistochemical, and stereological alterations in kidney tissue following prenatal CPF exposure, with assessments performed on postnatal day 28. In addition, we investigated the potential protective effects of *Ganoderma lucidum* (GNL), a medicinal mushroom known for its antioxidant and anti-inflammatory properties, as a biological strategy to mitigate organophosphate-induced developmental nephrotoxicity.

The observed increase in relative kidney weight, together with elevated serum urea and creatinine levels in CPF-exposed offspring, provides clear evidence of renal dysfunction and supports the nephrotoxic potential of prenatal CPF exposure. These findings are consistent with previous experimental studies reporting impaired renal function parameters and altered serum biochemistry following CPF administration [38,39]. Notably, GNL treatment significantly attenuated these alterations, suggesting a protective role in preserving renal function. In agreement with our findings, Zhong et al. demonstrated that *Ganoderma lucidum* polysaccharide peptide (GLPP) reduced renal dysfunction, improved histomorphological integrity, and decreased serum creatinine levels in experimental kidney injury models [19]. Similar renoprotective effects of GNL polysaccharides have been reported, including reductions in proteinuria and improvements in creatinine levels [40].

Collectively, these data suggest that GNL may counteract CPF-induced renal impairments through its antioxidant, anti-inflammatory, and cytoprotective properties. At the cellular level, GNL has been reported to mitigate toxic injury by stabilizing glomerular filtration dynamics and preserving nephron integrity. Moreover, modulation of oxidative stress-related and apoptosis-associated processes may contribute to its protective capacity to protect renal structure and function.

Biochemical analyses in the present study further support this interpretation. Elevated levels of malondialdehyde (MDA) and nitric oxide (NO), accompanied by marked reductions in superoxide dismutase (SOD), catalase (CAT), glutathione peroxidase (GPx), and reduced glutathione (GSH) in the CPF group, indicate a pronounced oxidative stress environment. Organophosphate compounds, including CPF, are known to disrupt antioxidant defense systems by increasing reactive oxygen species production, thereby promoting cellular damage [7]. Sustained oxidative stress has been shown to impair mitochondrial membrane potential, trigger cytochrome c release, and activate pro-apoptotic signaling cascades involving Bax and caspase-9/3 pathways. Indeed, CPF has been reported to modulate apoptosis and autophagy via multiple regulatory axes, including miR-19a–AMPK signaling, mTOR suppression, and p53 activation [41,42,43]. Furthermore, inhibition of the Nrf2/ARE pathway by CPF has been documented, leading to reduced cellular resilience against oxidative stress [44,45]. In this context, the decreased antioxidant enzyme activities observed in our study may reflect disruption of Nrf2-mediated cytoprotective regulation.

Consistent with this mechanism, numerous studies have demonstrated that GNL and its bioactive components reduce ROS accumulation, restore redox balance, and improve biochemical indices in experimental kidney injury models [19,46]. Triterpenes and polysaccharides derived from *Ganoderma lucidum* have been shown to exert potent antioxidant and anti-inflammatory effects, thereby mitigating oxidative injury across diverse renal pathologies. Accordingly, our findings suggest that GNL supplementation suppresses oxidative stress and facilitates restoration of biochemical homeostasis by counteracting CPF-induced increases in MDA and NO and preserving antioxidant defense capacity.

In addition to redox imbalance, the present study provides direct evidence of sustained inflammatory activation following prenatal CPF exposure. Renal TNF-α and IL-6 levels were markedly elevated in CPF-exposed offspring, indicating amplification of pro-inflammatory signaling pathways at postnatal day 28. Consistent with these findings, previous experimental work demonstrated that CPF exposure increases renal inflammation, nuclear factor-κB (NF-κB) activation, and pro-inflammatory cytokines such as TNF-α in rat kidneys [47].

Persistent cytokine elevation is known to exacerbate oxidative stress, disrupt mitochondrial integrity, and promote apoptotic cascades in renal tissue, as observed in various renal injury models [48]. In previous experimental studies, CPF-induced inflammatory signaling has been associated with activation of NF-κB and increased downstream cytokine production in renal tissue [49]. Although NF-κB activity was not directly assessed in the present study, the observed elevation in TNF-α and IL-6 levels suggests enhanced inflammatory signaling following prenatal CPF exposure. Importantly, GNL supplementation significantly attenuated TNF-α and IL-6 levels, supporting its anti-inflammatory potential in addition to its antioxidant effects, as oxidative stress modulators have been previously shown to suppress CPF-related inflammatory responses in the kidney [47]. These results support the notion that CPF-induced nephrotoxicity involves an inflammatory component in addition to oxidative stress-mediated injury.

Histopathological evaluations corroborated these biochemical findings. Prenatal CPF exposure resulted in pronounced renal tubular degeneration, epithelial cell loss, and interstitial alterations, accompanied by increased fibrosis and basement membrane thickening, as evidenced by H&E, Masson’s trichrome, and PAS staining. These observations are in line with previous reports demonstrating that CPF accelerates tubular degeneration, glomerular alterations, and fibrotic remodeling in renal tissue [4,6,50]. In contrast, GNL treatment markedly preserved renal histoarchitecture, reducing tubular injury and fibrotic changes. This protective effect may be attributed to the antioxidant capacity of GNL, its potential anti-inflammatory action, and its reported antifibrotic properties mediated through suppression of the TGF-β/Smad signaling pathway [19,46,51].

Apoptotic regulation represents another critical mechanism underlying CPF-induced nephrotoxicity. The balance between pro-apoptotic Bax and anti-apoptotic Bcl-2 proteins plays a decisive role in determining cellular fate. Bax promotes mitochondrial outer membrane permeabilization and cytochrome c release, whereas Bcl-2 counteracts this process. Accordingly, an increased Bax/Bcl-2 ratio reflects heightened susceptibility to apoptosis [52]. In the present study, immunohistochemical analyses revealed increased Bax expression, decreased Bcl-2 expression, and an elevated Bax/Bcl-2 ratio in the CPF group, consistent with enhanced apoptotic activity and renal tissue injury. Previous studies have similarly demonstrated that CPF activates mitochondrial apoptotic pathways and shifts the Bax/Bcl-2 balance toward a pro-apoptotic profile [45,53]. Protective agents, such as berberine, have been shown to mitigate CPF-induced renal injury through upregulation of Bcl-2 and downregulation of Bax, supporting the relevance of this pathway [54]. In line with these findings, GNL treatment in our study restored the Bax/Bcl-2 balance toward an anti-apoptotic profile, indicating attenuation of apoptotic signaling.

Beyond alterations in Bax and Bcl-2 immunoreactivity, quantification of cleaved (active) caspase-3 protein provided strong evidence of apoptotic pathway activation in renal tissue following prenatal CPF exposure. CPF-exposed offspring exhibited significantly elevated cleaved caspase-3 levels compared to controls, indicating execution-phase apoptosis rather than mere initiation of the death cascade. These findings are consistent with experimental models showing increased caspase-3 expression in the kidney following organophosphate-induced damage and apoptotic signaling activation [47].

Caspase-3 has been established as a pivotal regulator of apoptotic cell death in renal injury, with enhanced expression correlating with epithelial cell loss and tubular degeneration in acute kidney injury models [55]. Furthermore, organophosphate exposure has been shown to increase caspase-3 activity in both hepatic and renal tissues, supporting the notion that CPF disrupts mitochondrial integrity and drives programmed cell death via caspase-dependent pathways [56]. Notably, GNL administration significantly reduced cleaved caspase-3 levels relative to the CPF group, suggesting mitigation of execution-phase apoptotic signaling and preservation of renal cellular integrity.

The elevation of tubular injury biomarkers KIM-1 and NGAL in the present study provides compelling molecular evidence that CPF-induced biochemical and apoptotic alterations culminate in structural tubular damage. Kidney injury molecule-1 (KIM-1) and neutrophil gelatinase-associated lipocalin (NGAL) have been widely recognized as sensitive biomarkers of renal tubular injury, with their expression markedly increased in diverse nephrotoxicity models involving toxic and ischemic insults [57].

Previous experimental studies in rats have demonstrated that both KIM-1 and NGAL levels are significantly elevated in kidney tissue following nephrotoxic challenges, and their changes correlate with histopathological evidence of tubular damage [58]. Specifically, gentamicin-induced nephrotoxicity was associated with substantial upregulation of renal KIM-1 and NGAL, measured via ELISA and immunohistochemistry, validating their utility as early indicators of proximal tubular injury. Additionally, KIM-1 and NGAL have been documented to rise in rat kidney injury models, further supporting their role as reliable indicators of tubular compromise [59].

In agreement with these reports, CPF exposure in the current study resulted in significant increases in both KIM-1 and NGAL levels, consistent with sustained tubular epithelial injury. The significant reduction in these biomarkers upon GNL administration suggests that GNL treatment mitigates structural damage at the tubular level, aligning with its observed effects on upstream oxidative stress and apoptotic signaling.

Stereological evaluation using the Cavalieri principle further provided unbiased quantitative support for these structural alterations. In the present study, prenatal CPF exposure resulted in significant increases in cortical and glomerular volumes together with elevated relative kidney weight. Although volumetric enlargement might initially suggest enhanced growth, experimental studies indicate that renal and cortical volume increases frequently accompany pathological conditions such as inflammatory edema, vascular congestion, tubular dilation, and hypertrophic remodeling rather than preserved tissue integrity [60,61]. Indeed, stereological investigations in metabolic and toxic injury models have reported renal hypertrophy and cortical expansion concurrent with structural damage and oxidative stress [62]. Therefore, the volumetric changes observed in the CPF group likely reflect toxic injury-associated remodeling processes. Notably, *Ganoderma lucidum* co-administration attenuated these stereological alterations, suggesting preservation of renal architectural organization in parallel with the observed biochemical improvements.

At the developmental level, CPF has been shown to interfere with transcriptional networks regulating kidney development, including lncRNA–mRNA interactions, and to disrupt key nephrogenic signaling pathways such as Notch2–Jagged1 during embryogenesis. Such disturbances can lead to persistent structural and functional consequences in the postnatal period [36,63]. The observed reductions in cortical and glomerular volumes in our study are biologically consistent with this framework. CPF-induced oxidative stress and alterations in apoptotic markers (ROS ↑, MDA/NO ↑, Bax ↑/Bcl-2 ↓, cleaved caspase-3 ↑) are consistent with activation of the intrinsic apoptotic pathway and may accelerate epithelial cell loss, which is quantitatively reflected as diminished renal volume in stereological assessments [64,65].

Conversely, *Ganoderma lucidum* supplementation preserved cortical and glomerular volumes, suggesting maintenance of nephron integrity. Previous studies have demonstrated that GNL components protect renal tissue by reducing oxidative stress, stabilizing mitochondrial function, and suppressing fibrotic remodeling, primarily via modulation of the TGF-β/Smad axis [66]. These mechanisms are consistent with the stereological and histological preservation observed in the GNL-treated group.

The renal alterations observed at postnatal day 28 may have implications beyond the early postnatal period. Experimental and epidemiological evidence indicates that adverse intrauterine environments can induce persistent changes in kidney development, particularly through reduced nephron endowment and maladaptive structural remodeling [49,67]. A lower nephron number has been associated with compensatory hyperfiltration, glomerular hypertrophy, and increased susceptibility to progressive renal injury later in life [11]. Furthermore, emerging data suggest that early-life renal insults may predispose to chronic kidney disease through mechanisms involving persistent oxidative stress, mitochondrial dysfunction, and epigenetic modifications [68,69]. Although overt renal failure was not present at postnatal day 28 in the current study, the structural and molecular alterations detected may represent early events within a developmental programming trajectory that could increase vulnerability to chronic kidney disease in adulthood. Long-term follow-up studies are required to determine whether these early changes translate into functional impairment later in life.

The inclusion of only male offspring in the present study was based on well-documented sex-related differences in susceptibility to renal injury. Clinical and experimental evidence indicates that males exhibit a faster progression of chronic kidney disease compared to females [70]. Experimental studies further demonstrate that females display relative renoprotection, largely attributed to the antioxidant and antifibrotic effects of estrogen [71,72,73,74]. Therefore, the use of male offspring was intended to provide a sensitive and homogeneous model for detecting developmental nephrotoxic alterations. Nevertheless, given the regulatory role of sex hormones in oxidative stress and inflammatory pathways, extrapolation of these findings to female offspring should be approached with caution. Future studies including both sexes are warranted to clarify potential sex-dependent differences in developmental nephrotoxicity.

**Figure 13:**
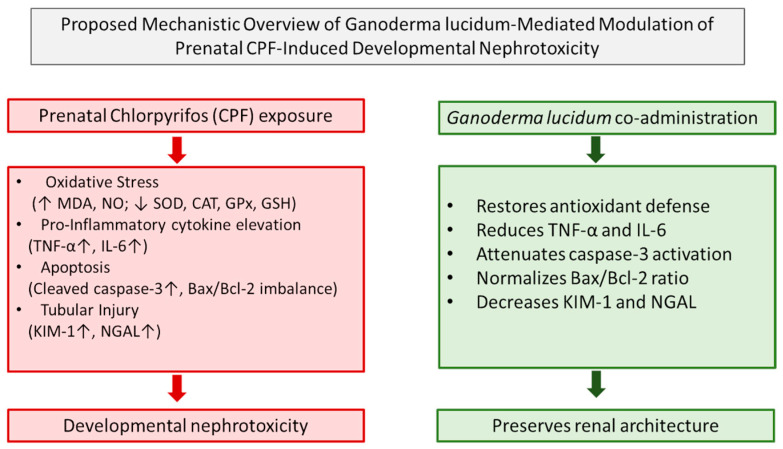
Proposed mechanistic overview of *Ganoderma lucidum*-mediated modulation of prenatal chlorpyrifos-induced developmental nephrotoxicity. Prenatal CPF exposure induced oxidative stress (↑ MDA, NO; ↓ SOD, CAT, GPx, GSH), increased pro-inflammatory cytokines (↑ TNF-α, IL-6), activated mitochondrial apoptotic signaling (↑ cleaved caspase-3; Bax/Bcl-2 imbalance), and elevated tubular injury biomarkers (↑ KIM-1, NGAL), leading to structural and stereological renal alterations in offspring kidneys. *G. lucidum* co-administration attenuated these interconnected pathways and preserved renal architecture. Red boxes and arrows indicate CPF-induced pathological processes, whereas green boxes and arrows represent the protective effects of *G. lucidum*. Upward arrows (↑) indicate increased levels, and downward arrows (↓) indicate decreased levels of the respective markers.

Taken together, the present findings demonstrate that prenatal CPF exposure induces developmental nephrotoxicity through an integrated cascade involving oxidative stress, inflammatory amplification, mitochondrial apoptotic activation, and tubular injury. The concurrent elevation of TNF-α, IL-6, cleaved caspase-3, KIM-1, and NGAL provides molecular confirmation that CPF-mediated redox imbalance translates into sustained inflammatory signaling, execution-phase apoptosis, and structural renal damage. Conversely, *Ganoderma lucidum* exerts a coordinated renoprotective effect by modulating oxidative stress, suppressing inflammatory cytokine production, attenuating caspase-3 activation, and preserving tubular architecture (Figure 13).

## 5. Limitations

The present study focused on evaluating biochemical, structural, and functional alterations associated with prenatal CPF-induced developmental nephrotoxicity and their modulation by *Ganoderma lucidum*. Although inflammatory cytokines (TNF-α and IL-6), cleaved caspase-3, and tubular injury biomarkers were quantitatively assessed, detailed upstream molecular signaling analyses were not included in the current experimental framework. Therefore, the proposed involvement of signaling pathways such as Nrf2/ARE, NF-κB, and TGF-β/Smad is based on previously published experimental evidence rather than direct measurement within the present study. Nevertheless, the integration of oxidative stress parameters, antioxidant enzyme activities, inflammatory mediators, apoptotic indicators, histopathology, and unbiased stereological quantification provides consistent and multi-level evidence supporting the proposed pathogenic cascade. Future studies incorporating advanced molecular approaches are required to directly verify these upstream regulatory mechanisms and further clarify the signaling networks involved.

## 6. Conclusions

The present study demonstrates that prenatal chlorpyrifos (CPF) exposure induces persistent developmental nephrotoxicity characterized by functional impairment, redox imbalance, inflammatory amplification, apoptotic activation, and structural renal alterations in offspring evaluated at postnatal day 28. CPF exposure was associated with increased oxidative stress markers, elevated TNF-α and IL-6 levels, enhanced cleaved caspase-3 expression, and a shift in Bax/Bcl-2 balance, indicating activation of intrinsic apoptotic signaling. These molecular alterations were accompanied by significant elevations in tubular injury biomarkers (KIM-1 and NGAL) and stereologically confirmed reductions in cortical and glomerular volumes, reflecting compromised nephron integrity.

Conversely, *Ganoderma lucidum* supplementation attenuated oxidative stress, suppressed inflammatory cytokine production, reduced caspase-3 activation, improved Bax/Bcl-2 balance, and lowered KIM-1 and NGAL levels, resulting in preservation of renal histoarchitecture and morphometric parameters. Collectively, these findings suggest that CPF-induced renal injury involves interconnected oxidative, inflammatory, and apoptotic processes, while GNL exerts coordinated renoprotective effects through modulation of these pathways.

These results highlight the potential long-term renal consequences of prenatal organophosphate exposure and support further investigation into antioxidant and anti-inflammatory strategies for mitigating developmental nephrotoxicity.

## Figures and Tables

**Table 1:** Stereological Sampling Parameters and Coefficient of Error (CE) Values.

	Sections per Animal (Mean ± SD)	Points Counted per Animal (Mean ± SD)	CE (Mean ± SD)
**Cortex**	15.0 ± 1.31	1008.75 ± 4.80	0.004 ± 0.001
**Medulla**	15.63 ± 1.19	1011.38 ± 8.13	0.004 ± 0.001
**Glomerulus**	15.38 ± 1.60	984.38 ± 44.36	0.019 ± 0.028

## Data Availability

The data presented in this study are available from the corresponding author upon request.
